# Noninvasive Diagnosis of Visceral Leishmaniasis: Development and Evaluation of Two Urine-Based Immunoassays for Detection of *Leishmania donovani* Infection in India

**DOI:** 10.1371/journal.pntd.0005035

**Published:** 2016-10-14

**Authors:** Sarfaraz Ahmad Ejazi, Pradyot Bhattacharya, Md. Asjad Karim Bakhteyar, Aquil Ahmad Mumtaz, Krishna Pandey, Vidya Nand Ravi Das, Pradeep Das, Mehebubar Rahaman, Rama Prosad Goswami, Nahid Ali

**Affiliations:** 1 Infectious Diseases and Immunology Division, Indian Institute of Chemical Biology, Kolkata, India; 2 Department of Medicine, Shri Krishna Medical College and Hospital, Muzaffarpur, India; 3 Department of Clinical Medicine, Rajendra Memorial Research Institute of Medical Sciences, Patna, India; 4 Department of Molecular Biology, Rajendra Memorial Research Institute of Medical Sciences, Patna, India; 5 Department of Tropical Medicine, School of Tropical Medicine, Kolkata, India; Academic Medical Centre, NETHERLANDS

## Abstract

**Background:**

Visceral Leishmaniasis (VL), a severe parasitic disease, could be fatal if diagnosis and treatment is delayed. Post kala-azar dermal leishmaniasis (PKDL), a skin related outcome, is a potential reservoir for the spread of VL. Diagnostic tests available for VL such as tissue aspiration are invasive and painful although they are capable of evaluating the treatment response. Serological tests although less invasive than tissue aspiration are incompetent to assess cure. Parasitological examination of slit-skin smear along with the clinical symptoms is routinely used for diagnosis of PKDL. Therefore, a noninvasive test with acceptable sensitivity and competency, additionally, to decide cure would be an asset in disease management and control.

**Methodology/principal findings:**

We describe here, the development of antibody-capture ELISA and field adaptable dipstick test as noninvasive diagnostic tools for VL and PKDL and as a test of cure in VL treatment. Sensitivity and specificity of urine-ELISA were 97.94% (95/97) and 100% (75/75) respectively, for VL. Importantly, dipstick test demonstrated 100% sensitivity (97/97) and specificity (75/75) in VL diagnosis. Degree of agreement of the two methods with tissue aspiration was 98.83% (κ = 0.97) and 100% (κ = 1), for ELISA and dipstick test, respectively. Both the tests had 100% positivity for PKDL (14/14) cases. ELISA and dipstick test illustrated treatment efficacy in about 90% (16/18) VL cases when eventually turned negative after six months of treatment.

**Conclusions/significance:**

ELISA and dipstick test found immensely effective for diagnosis of VL and PKDL through urine samples thus, may substitute the existing invasive diagnostics. Utility of these tests as indirect methods of monitoring parasite clearance can define infected versus cured. Urine-based dipstick test is simple, sensitive and above all noninvasive method that may help not only in active VL case detection but also to ascertain treatment response. It can therefore, be deployed widely for interventions in disease management of VL particularly in poor resource outskirts.

## Author Summary

Visceral leishmaniasis (VL), one of the most prevalent parasitic diseases in the developing world causes serious health concerns. Post kala-azar dermal leishmaniasis (PKDL) is a skin disease which occurs after treatment as a sequel to VL. Parasitological diagnosis involves invasive tissue aspiration which is tedious and painful. Commercially available immunochromatographic rapid diagnostic test such as rK39-RDT is used for field diagnosis of VL, detects antibodies in serum samples. Urine sample is however, much easier in collection, storage and handling than serum and would be a better alternative where collection of tissue aspirate or blood is impractical. In this study, we have developed and evaluated the performance of two urine-based diagnostic assays, ELISA and dipstick test, and compared the results with serological rK39-RDT. Our study shows the capability of urine-based tests in detecting anti-*Leishmania* antibodies effectively for both VL and PKDL diagnosis. The ability of dipstick test to demonstrate negative results after six months in 90% of the VL cases after treatment could be useful as a test of clinical cure. Urine-based tests can therefore replace the need for invasive practices and ensure better diagnosis under filed settings.

## Introduction

Visceral Leishmaniasis (VL) is a vector-borne fatal infectious disease disseminated in 88 countries of the world, particularly in remote areas of India, Bangladesh, Sudan, Brazil, Ethiopia and South Sudan where 90% of the cases occur [1]. Around 200 to 400 thousands of new VL cases are reported every year globally [2]. Definitive diagnosis is important in the early phase of infection as drugs available for this disease have severe side effects [3]. Microscopic visualisation of spleen or bone marrow aspirates considered as a gold standard test for the confirmation of VL is conventionally used wherever feasible, although risky, painful and an invasive practice demanding expertise [4]. A molecular diagnosis like polymerase chain reaction (PCR) is constrained to research labs and tertiary hospitals only [5]. The presence of high levels of antibodies in the sera of VL patients was exploited for serological diagnosis using enzyme-linked immune sorbent assay (ELISA), direct agglutination test (DAT) and immunochromatographic rapid diagnostic test (RDT). The fact that ELISA and DAT are time taking, sophisticated and labour intensive, limits these methods for routine diagnosis in the majority of the VL endemic areas [6]. Antigen rk39 (39 amino acid kinesin-related protein) based RDTs are the most commonly used rapid test for sero-diagnosis of VL, especially in the Indian subcontinent where it gave sensitivity and specificity estimates of 97% and 90.2%, respectively [7]. However, their diagnostic performance varies from moderate in Latin America to even low in East African region [8]. Other than rK39, many newer recombinant antigens such as rKE16, rK28, and rKLO8 have been developed in the last decade for serological diagnosis [9–11]. *Leishmania*-specific antibodies remain within the infected blood for years after treatment. Thus serum-based antibody detection tests cannot serve as a test of cure [12].

To overcome the shortcomings of serum-based diagnostic tests several studies have been conducted to find a noninvasive biological source for VL diagnosis such as use of saliva and urine samples instead of serum [13]. For example, PCR have been found 88%-96.8% sensitive when conducted with DNA extracted from urine samples [14–16]. Saliva as diagnostic sample has been reported with 58.6%-82.5% and 83.3% sensitivity in rK39-RDTs and ELISA, respectively [17,18]. rK39-RDTs for sero-diagnostics were also tested with urine samples in several studies and found sensitivities and specificities of 96.40% and 66.2–100%, respectively [19]. Urine-based latex agglutination test (KAtex) for detecting leishmanial antigen in urine is hampered by low sensitivity (47%-95%) and obligation to boil the samples to increase the test specificity [20]. Apart from KAtex, several urine-based antigen detection assays have been reported in recent years with varying performances [21,22]. Very few antibody capture ELISA have been conducted for diagnosis of VL through urine samples but not in the Indian region [23,24].

Post kala-azar dermal leishmaniasis (PKDL) is a skin disease caused by the same *Leishmania donovani* parasite responsible for VL [25]. In VL endemic areas serology with clinical presentation such as macules, papules, plaques or nodules, suggest probable PKDL. However, confirmation of PKDL relies on parasitological examination of slit-skin smear (SSS) or biopsy [26].

In the present study, we aimed to develop and evaluate urine-based ELISA and dipstick test as noninvasive diagnostic tools and compared with serum-based commercially available rK39-RDT using parasitologically confirmed VL cases from geographically important endemic areas of India. We have also assessed the performance of ELISA and dipstick test for PKDL diagnosis through urine samples. To determine the presence of VL antibodies of urine in response to therapy, this study was extended to comprise the evaluation of urine-based ELISA and dipstick test after six months of treatment. Our results confirmed the potential of urine samples in ELISA and dipstick test for the noninvasive diagnosis of VL and PKDL and as a test of clinical cure for human VL.

## Methods

### Sample collection

A total of 186 participants were enrolled in the School of Tropical Medicine (STM), Kolkata, Rajendra Memorial Research Institute of Medical Sciences (RMRIMS), Patna, Shri Krishna Medical College and Hospital (SKMCH), Muzaffarpur and Indian Institute of Chemical Biology (IICB), Kolkata during March 2011 to July 2016. Urine samples of 97 parasitologically proven and rK39-RDT (InBios Int. Inc., USA) tested VL cases were obtained before the treatment started. Out of 97 VL samples, 18 were acquired for longitudinal study at different time points, before treatment began (day 0), when the treatment completed (day 30), and after six months since the first sample collected (day>180). Samples were also collected from 75 control subjects including 25 non-endemic healthy controls (NEHC) from IICB, 25 endemic healthy controls (EHC) from RMRIMS and STM and 25 symptomatically similar diseases other than VL (OD) from STM. Fourteen urine samples which were confirmed by skin biopsy or slit-skin smear were collected from PKDL patients. Immediately after collection of urine, sodium azide (NaN_3_) was added to each sample at a final concentration of 0.1% as a preservative. The samples were stored at 4°C refrigeration until use.

### Ethics statement

This study was approved by the Ethical Committee of Indian Institute of Chemical Biology, Kolkata and by the respective hospitals. Written informed consent in their local languages was taken from the participants or parents, if not adult prior to the study. The reason of collection and storage of urine samples for evaluation of newly developed immunoassays was clearly mentioned in the consent form.

### ELISA

*L*. *donovani* strain AG83 (ATCC PRA-413) parasite was cultured according to the standard protocol [27]. Recombinant proteins of strain AG83 namely, rGP63 (a 63 kDa recombinant glycoprotein) and rCPA (a recombinant cysteine protease) were purified from its clones [28,29]. Soluble leishmanial antigens (SLA) extracted from *L*. *donovani* were prepared by the following [30]. *Leishmania* promastigote membrane antigen (LAg) was extracted as described [31]. Antibody capture ELISA was performed using 96-well flat bottom ELISA plates (Nunc Maxisorp, Denmark). Briefly, 1 μg/well of LAg in phosphate buffer was coated, and plates were kept overnight at 4°C, afterwards blocked with 1% of bovine serum albumin (BSA) (Sigma, USA) in phosphate buffer saline (PBS) for 2 h at 37°C. Subsequently, 1:10 dilution of urine samples followed by 1:4000 diluted HRP-conjugated anti-human IgG (Bangalore GeNei, India) were applied and incubated at 37°C for 1 h. Plates were washed between each step with PBS containing Tween-20. Finally, 5 mg o-phenylenediamine dihydrochloride (OPD, Sigma, USA) and 0.05% H_2_O_2_ (Merck, Germany) in 10 ml phosphate-citrate buffer were added for 15 min at room temperature (RT) and the optical densities were read at 492 nm.

### Dot blot assay

Nitrocellulose membrane (Hybond 0.45 μm, GE Healthcare, UK) was cut in the form of rectangular strips and soaked in 25 mM Tris-HCl buffer (pH 7.6). In a semi-dried condition, 1.5 μg of LAg in 2 μl Tris-HCl buffer were coated in the form of a dot. After complete drying, free areas of the strips were blocked with 5% of BSA + 0.1% Tween-20 and 0.01% NaN_3_ in 100 mM Tris Buffer Saline (TBS) and incubated at 4°C overnight. Next day, strips were washed thrice with 100 mM TBS and 0.05% Tween-20 (TBS-T) followed by drying at RT. Subsequently, urine samples at 1:5 dilution in TBS-T were incubated with the strips for 30 min at RT. After two washes in TBS-T, strips were incubated with 1:2000 diluted peroxidase-conjugated goat anti-human IgG for 30 min at RT. Following, two washes in TBS-T and a final wash in 100 mM TBS (without Tween-20), strips were incubated for 5 min in a freshly prepared substrate solution having 0.05% of 3, 3’-diaminobenzidine tetrahydrochloride (DAB, Sigma, USA) containing 0.05% of H_2_O_2_ in 100 mM TBS. The reaction was stopped by dipping the strips in distilled water for 2 min. Dark brown colour spot depicted anti-*Leishmania* reactivity of urine samples.

### Dipstick development and assay

Nitrocellulose membrane-based dipstick was prepared to have a test and a control line for visual detection of the disease. In brief, 1.5 μg of LAg/dipstick at the test line and 1: 20 dilution of rabbit anti-human IgG at the control line were bound to the membrane and blocked with 5% BSA at 4°C overnight. Next day after drying, the membrane was adhered to a plastic sheet and stored at RT until the test. Like dot blot assay the dipstick test comprises incubation with urine sample followed by enzyme-conjugated anti-human IgG and then substrate chromogen including washing in each step. Dark brown coloured bands both at the test and control line show VL positivity and a single band at the control line infers VL negativity.

### Statistics

Statistical analyses were performed with Graph Pad Prism version 5.0. Two-tailed, Mann-Whitney *U* test was used for comparison of ELISA values of different groups and considered statistically significant if the *P* values<0.05. A receiver-operator characteristic (ROC) curve for ELISA was constructed to determine the cut-off value with 95% confidence intervals (CI) that discriminate between VL-positive and -negative urine samples. To assess the overall performance of the test sensitivity and specificity were calculated and diagnostic accuracy was established by area under the curve (AUC) where AUC = 1 indicates an accurate test. Kappa values (0.8–1, perfect agreement) were estimated to find the degree of agreement for urine-based ELISA and dipstick test with the reference test of tissue aspiration.

## Results

### Antibody capture ELISA for diagnosis of VL and PKDL

**Fig 1:**
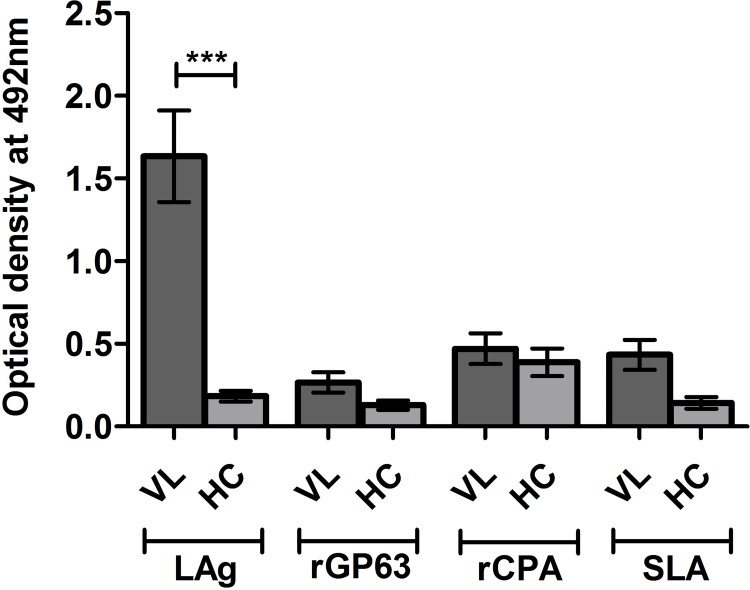
Comparison of urine antibody absorbances between VL patients and healthy controls using four leishmanial antigens. Reactivity of urine (undiluted) from visceral leishmaniasis (VL) patients (n = 5) and healthy controls (HC) (n = 6) against 2.5 μg/well of leishmanial membrane antigen, LAg; recombinant glycoprotein 63, rGP63; recombinant cysteine protease A, rCPA and soluble leishmanial antigen, SLA. The standard error of mean for each group is shown as a solid line.

**Fig 2:**
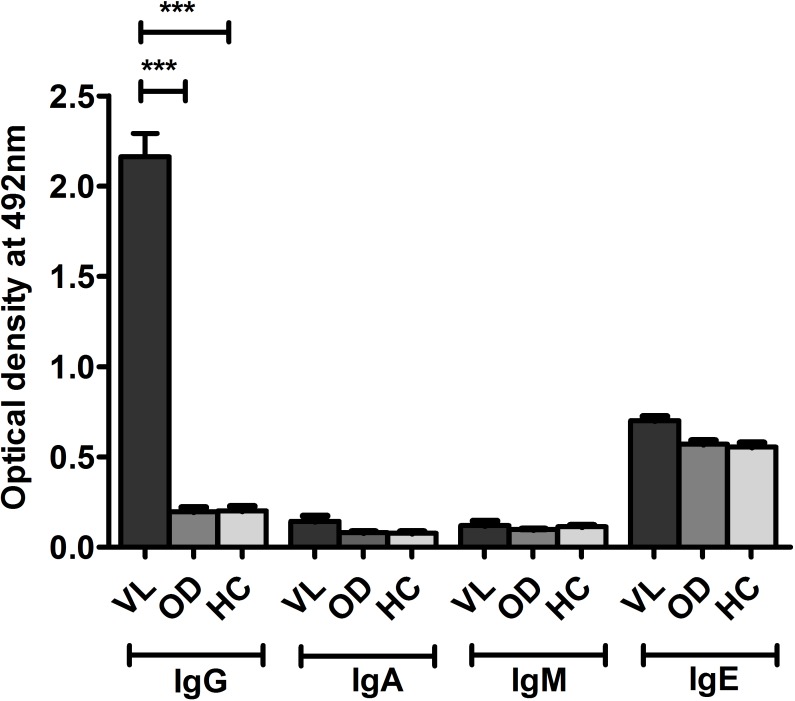
ELISA to determine relative antibody isotype titres in urine of VL patients and negative controls. Graph showing comparative optical density values of LAg-specific antibody isotypes; IgG, IgA, IgM, and IgE present in the urine samples of VL patients (n = 5), healthy controls (HC) (n = 3) and other diseases (OD) (n = 4) at 492nm.

**Fig 3:**
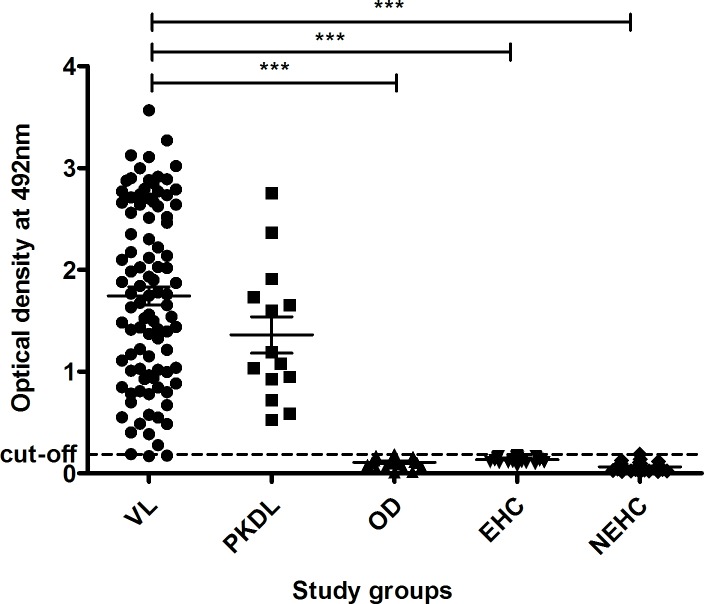
ELISA results with urine samples obtained from VL and PKDL patients, healthy individuals and other clinical groups. Comparative evaluation of ELISA reactivity of anti-*Leishmania* antibodies in urine against LAg at 0.1875 cut-off. The study groups were composed of a panel of VL patients (VL; n = 97), PKDL patients (n = 14), endemic healthy controls (EHC; n = 25), non-endemic healthy controls (NEHC; n = 25) and other diseases (OD; n = 25) including malaria (n = 2), viral fever (n = 2), tuberculosis (n = 4), typhoid (n = 4), liver abscess (n = 3), systemic lupus erythematosus (SLE) (n = 3), sepsis (n = 2), pancreatitis (n = 1), pneumonia (n = 1), filariasis (n = 1), abdominal pain and loose motion (n = 1), and mouth ulcer (n = 1). Each sample was tested in duplicate and the mean was taken. Each dot represents mean of a single sample.

**Fig 4:**
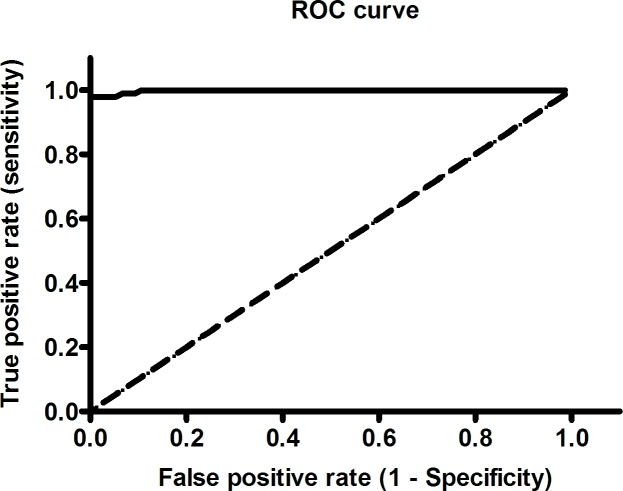
Receiver operator characteristics (ROC) curve for urine ELISA. ROC curve obtained from the ELISA values for the detection of anti-*Leishmania* IgG antibodies in urine samples. Cut-off value (0.1875), sensitivity (97.94%), specificity (100%) and AUC (0.9984) were determined by this curve using GraphPad Prism software (version 5.0).

To assess *Leishmania* proteins as potential noninvasive diagnostic candidate antibody capture ELISA was performed as a proof of principle experiment with four leishmanial antigens, rGP63, rCPA, SLA and LAg. LAg showed better recognition of IgG antibodies in VL urine with high specificity compared to other tested antigens (Fig 1). Reactivity of LAg was also investigated against other antibody isotypes, IgA, IgM and IgE present in the urine samples. Presence of high levels of anti-leishmanial IgG antibodies in VL urine samples than non-VL controls distinguishes disease condition better over other antibody isotypes (Fig 2). Parameters such as urine dilution and antigen concentrations were standardised to set optimal ELISA condition for the diagnosis (S1 and S2 Figs). Finally, all urine samples were examined by ELISA and results were interpreted to find urine IgG reactivity of each group against LAg (Fig 3). With the assessment of ROC curve (Fig 4), the test cut-off point, 0.1875 was selected from possible cut-offs at 95% CI. Urine from VL patients illustrated significantly (*P* <0.0001) stronger recognition against LAg with a high degree of specificity. The test showed positivity in 95 of 97 VL cases, yielding a sensitivity of 97.94% for detection of *Leishmania* exposure. False negative results were found for 2 (2.06%) of 97 VL samples. All 14 PKDL samples showed antibody titres above the cut-off line thus 100% sensitive for urine ELISA. Overall specificity was calculated using 75 non-VL urine samples of controls and found 100% specificity without any false positives. Anti-LAg antibody titre in VL urine is 27, 12 and 16 times higher than those of NEHC, EHC and OD, respectively (Fig 3). The AUC obtained for the test was 0.9984, depicting its potential to discriminate cases with and without the disease and comprises a high degree of agreement (κ = 0.97) with the gold standard test. Additionally, two VL cases which were parasitologically confirmed but negative to rK39-RDT with serum were found positive in urine ELISA, suggesting their better performance in comparison to serum rK39-RDT.

### Urine ELISA as a test of cure

**Fig 5:**
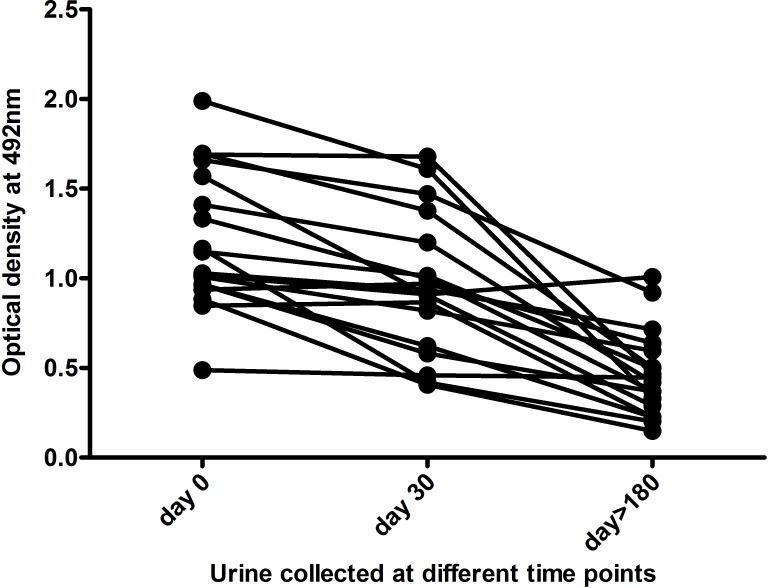
Comparison of urine antibody titres of VL patients at different time points of treatment. Absorbance value of LAg-specific urine antibody titres in VL at day 0, before the treatment started; at day 30, after drug therapy and at days >180, more than 6 months of treatment started.

To determine the urine antibody levels in response to treatment, we selected paired urine samples from 18 VL patients at day 0, day 30 and day >180. There is no significant decline in the titre just after completing the treatment (day 30). Out of 18 urine samples, 16 showed a significant decrease (p<0.0001) in the urine IgG titre only after six months (day > 180) since the treatment started (Fig 5).

### Dot blot assay

**Fig 6:**
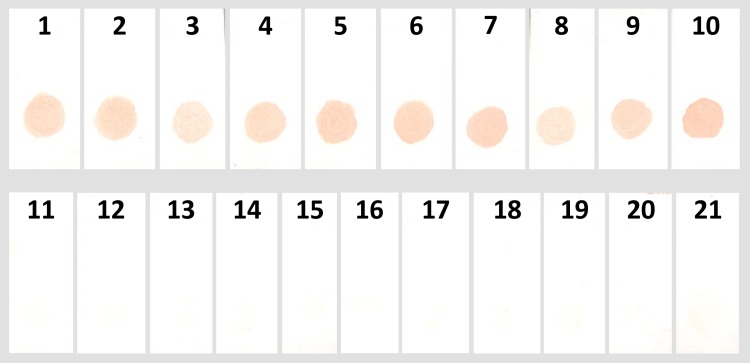
Dot blot assay to identify *L*. *donovani* infection. Results showing dot blot assay with VL; 1–10, malaria; 11 and 12, viral fever; 13 and 14, tuberculosis; 15 and 16, typhoid; 17, endemic healthy control; 18 and 19 and non-endemic healthy control; 20 and 21.

Principles of ELISA were adapted in the form of dot blot to develop a nitrocellulose membrane based diagnostic test. Dot blot optimisation was performed with urine samples from different groups to see the qualitative reactivity of the antigen on the membrane. Antigen concentration of 1.5 μg/dot, urine dilution of 1:5 and blocking with 5% BSA were optimised for the assay (S3–S5 Figs). Under these conditions, 10 VL samples and 11 non-VL controls were used in the dot blot experiment. Urine from confirmed VL cases tested positive, and the non-VL controls were all negative in the assay as determined by the visual observation (Fig 6). Anti-human IgG was selected and optimised for the assay as an experimental control which show reactivity with urine IgG irrespective of their specificity (S6 Fig).

### Dipstick assay as a noninvasive diagnostics for VL and PKDL

**Fig 7:**
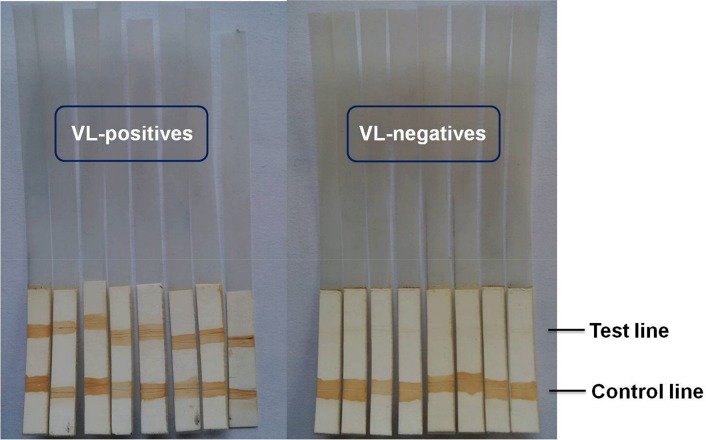
Representative image of dipstick immunochromatographic test. Dipstick assay shows positive results with double band at the test and control lines and negative results with bands only at the control line.

Dot blot assay was transformed into a field adaptable dipstick format as discussed in Methods Section for the qualitative detection of total IgG in human urine (S7 Fig). Results of the dipstick test showed positivity in all VL urine samples, acquiring a sensitivity of 100% (97/97). None of the urine samples taken from negative controls showed reactivity with the dipstick assay, resulting in 100% specificity (75/75) of the test. Thus, complete agreement (κ = 1.0) was observed between the dipstick test and the reference test of tissue aspiration for diagnosis of VL. In comparison to urine dipstick test, serum-based rK39-RDT showed 95.87% sensitivity (93/97) and 97.33% specificity (73/75). Two patients with confirmed VL, who were false negative in our ELISA and with serum rK39-RDT, demonstrated positive dipstick test showing its aptitude to detect even low antibody titre in urine. All 14 PKDL urine samples tested had positive results in dipstick assay thus showing 100% sensitivity of the test. The analytical performances of dipstick test and ELISA with rK39-RDT are compared in Table 1, and the representative illustrations of dipstick assay are shown in Fig 7.

**Table 1:** Sensitivity and specificity of urine immunological assays in comparison to serological rK39 test with 95% confidence interval.

	Serum			Urine					
Subjects (n)	rK39-RDT			LAg-ELISA			LAg-Dipstick test		
	Sensitivity N (%)	Specificity N (%)	95% Confidence Interval	Sensitivity N (%)	Specificity N (%)	95% Confidence Interval	Sensitivity N (%)	Specificity N (%)	95% Confidence Interval
**VL (97)**	**93 (95.87)**	NA	89.78–98.87	**95 (97.94)**	NA	92.75–99.75	**97 (100)**	NA	96.26–100
**PKDL (14)**	**14 (100)**	NA	95.20–100	**14 (100)**	NA	95.20–100	**14 (100)**	NA	95.20–100
**NEHC (25)**	NA	25 (100)	86.28–100	NA	25 (100)	86.28–100	NA	25 (100)	86.28–100
**EHC (25)**	NA	25 (100)	86.28–100	NA	25 (100)	86.28–100	NA	25 (100)	86.28–100
**OD (25)**	NA	23 (92)	73.97–99.02	NA	25 (100)	86.28–100	NA	25 (100)	86.28–100
**Total Controls (75)**	-	**73 (97.33)**	90.70–99.68	-	**75 (100)**	95.2–100	-	**75 (100)**	95.2–100

Comparative performances of urine-ELISA and Urine dipstick test with serum rK39 rapid diagnostic test

VL, visceral leishmaniasis

PKDL, post kala-azar dermal leishmaniasis

NEHC, non-endemic healthy controls

EHC, endemic healthy controls

OD, other diseases than VL

NA, not applicable.

### Dipstick assay as a test of cure

Evaluation of dipsticks as a test of cure was conducted with 18 paired VL samples at day 0, 30 and >180 after treatment initiation. Samples after one month of therapy (day 30) showed a decrease in the positivity only in 5 samples as the test line of the dipstick had less intense colour than the samples using before treatment started (day 0). Out of 18 follow-up samples, 16 were entirely negative on and after six months of the treatment initiation (day >180). Two samples remained positive after 6 months of treatment till the data reported (S1 Table).

## Discussion

Noninvasive diagnosis of VL has been a challenge for long, and to date no accepted urine-based rapid test is available. WHO in their 3^rd^ report on neglected tropical diseases in 2015 recommended the need of improved diagnostic tests for VL, PKDL and test of cure. We report here, ELISA and a field adaptable dipstick test which make use of urine samples instead of serum for diagnosis of VL as well as PKDL diseases. To the best of our knowledge, this is the first study where a dedicated dipstick test was developed for the detection of leishmanial antigen-specific antibodies from human urine samples and 100% sensitivity, and specificity was achieved. We have also reported an ELISA for the first time with urine of PKDL patients, where it gives 100% sensitivity. Additionally, ELISA and dipstick assay show clearance of the antibodies from 90% of the urine samples six months after start of treatment. Unlike the blood stream where antibodies persist for several years after treatment, our data show prospects in the use of these urine tests for monitoring prognosis following treatment.

Employing LAg as the antigen, the respective sensitivities and specificities were found to be 97.94% and 100% in ELISA, and 100% and 100%, in dipstick assay for VL. These findings are consistent with our earlier reports of diagnostic ability of LAg in detecting serum antibodies [32]. Single antigens so far were not found very beneficial for diagnosis of VL. LAg is a mixture of at least ten urine-reactive antigenic polypeptides present in the membrane of the parasite *L*. *donovani*. Reactivity of each polypeptide for urine antibodies gives synergistically better diagnostic performance than an antigen alone thus justifying its better diagnostic aptitude over cloned antigens.

Urine as diagnostic tool is a thrust in VL research. Several established serological methods such as ELISA, DAT and RDTs have been tested with urine samples in recent studies. For example, DAT when conducted with urine samples showed 90.7% and 96.4% sensitivity and specificity, respectively [33]. Attempts have been made in the past few years to identify protein biomarkers in urine during VL [34,35]. A latex agglutination test such as KAtex has suboptimal sensitivity in detecting urine antigen in VL diagnosis [20]. A prototype ELISA kit detecting urinary antigen was developed and evaluated in Ethiopia, Sudan, Bangladesh and Brazil. The overall sensitivities of the kit ranged from 88.4 to 100% with 100% specificity [21]. *Leishmania infantum* antigens which are excreted in urine of VL patients were also identified and cloned [36]. All VL urine samples were reported to be positive in capture ELISA when three leishmanial antigen assays were combined [22].

Antibody detection in urine could be beneficial for the noninvasive diagnosis of VL. However, only few were performed in this area. Islam *et al* in Bangladesh have investigated urine IgG using *L*. *donovani* crude antigen in ELISA and reported 93.5% sensitivity and 89.3% specificity [23]. The same group later showed 94% and 99.6% sensitivity and specificity, respectively with kinesin-related antigen rKRP42 [24]. In these studies VL follow-ups and PKDL patients were not included and the studies have been only at the ELISA level. Very recently, in Bangladesh Ghosh *et al* have observed only a marginal drop in the sensitivity and >96% specificity when urine samples were used and compared with serum by antibody-capture ELISA. In the study antigen rK28 demonstrated 95.4% sensitivity with urine than 98.9% with serum samples[37]. We have reported urine-ELISA for the first time with the Indian VL patients and the first to report for PKDL patients. Here the studies were conducted in three major centres of India which cover the key endemic areas of VL in this region.

There is no rapid test available for urine-antibody detection from VL and PKDL patients. Serum-based rK39-RDTs were tested with urine samples in a study in India and found very low specificity ranging from 66.2% to 77.08% only, thus not considered suitable for VL diagnosis in this format [38]. However, similar studies in Bangladesh and two other groups in India have shown sensitivities and specificities ranging from 95% to 100% and 86.33% to 100%, respectively [39–41]. Few studies also reported the reactivity of rK39-RDT with PKDL urine samples though the number of samples used was very few and the results were contradictory. One of our co-authors Goswami *et al* [40] showed PKDL positivity with rK39-RDT in six urine samples while Mohapatra *et al* [13] reported 3 out of 3 negative PKDL results with urine samples. We observed 100% urine positivity of PKDL cases (14/14) both in ELISA and dipstick tests. Diagnosis of PKDL is of utmost importance as it may harbour *Leishmania* parasites in the skin and provide another reservoir for *Leishmania* infection. However, PKDL is not a fatal infection and often evades VL surveillance programmes. Noninvasive diagnosis of PKDL can help in early identification of PKDL sufferers.

Except the invasive tissue aspiration, present serological diagnostic tests are not good enough for use as ‘test of cure’ [42]. Decline in antibody response against K39 antigen have been reported in VL patients but the time to become sero-negative varied [43]. A study showed antibodies against rK39 antigen remains unaltered in serum after 180 days of treatment. However, antibodies against rK26 and rK18 demonstrated significant decline in antibody titre of serum samples after 6 months of treatment [44]. Recently, urine antigen response after VL therapy has been investigated through ELISA. In the study, Leishmania Antigen Detect ELISA showed 100% negativity of urine sample at 180 days of treatment [21]. Antibody detection through serum-based rK39-RDT has been tested with urine of treated VL individuals in two separate studies. Urine samples of 40 VL follow-ups after 1–3 months and 11 urine samples just after treatment completion were positive with rK39-RDT thus demonstrating its failure as a test of cure [13,40]. We explored for the first time a significant (16/18) fall of urine antibody titre in ELISA after 180 days of treatment. Moreover, results of dipsticks tests which were positive before treatment turned completely negative (16/18) within six months of treatment. Positivity in two samples even after six months might be an indication of relapse in future and needs to be monitored for disease. Our results demonstrating the prognostic use of urine-based ELISA and dipstick assay warrant further detailed longitudinal studies.

Collection, storage and handling of urine samples are safe, noninvasive and advantageous over serum samples. The advantage of this is particularly to infants with *Leishmania* infection, from whom the collection of blood is difficult. Although, dried blood spot on filter paper has been proven successful in diagnosis and epidemiological studies of VL using DAT, however, DAT requires central laboratory facility [6,45]. Regarding urine dipstick test, it is a ready-to-use device which is stored at room temperature and can be used for at least one year with simple desiccation. It is also easier in handling and does not involve much expertise or sophisticated instruments, thus suitable for outreach diagnosis. The assay does not require pretreatment of the urine samples as in KAtex test. Urine samples were tested and found stable and reproducible up to more than six years when stored with 0.1% azide at 4°C [24].

This study, however, has limitations. The VL subjects chosen for the studies were clinically confirmed thus cannot give an idea about infection versus disease. Population-based study will be required to evaluate the performance of dipstick test with VL suspected individuals and asymptomatic carriers in defined VL endemic areas. Moreover, to compare treatment response, blood samples could not be collected from follow-up patients who came in the hospital outpatient department for routine checkup after at least 6 months of treatment. However, previous reports suggest persistence of anti-*Leishmania* antibodies up to six months of VL treatment [43,44]. This dipstick is currently based on enzyme-catalysed colorimetric reaction which takes 90 min to complete (S8 Fig). It could be reduced to 5 min with the existing gold-tagged lateral flow technology (already started).

Our findings highlight the potentials of urine-based ELISA and dipstick tests that can offer an efficient and convenient alternative to invasive diagnostics of VL as well as PKDL. Dipstick may help to overcome the need for invasive tissue aspiration particularly in primary health care centres where it is unlikely to be feasible.

Like in the Indian subcontinent, use of rK39-RDT with strict clinical case definition has marked a good impact in screening VL cases at the primary level. In East African regions where performance of rK39-RDT is not good this urine-based dipstick could be valuable in VL diagnosis at field settings. Moreover, dipstick test can also suggest the treatment response thus can be used as a test of cure. The collection of urine is comparatively easier and painless than withdrawing blood so it can help to screen *Leishmania* exposure at remote areas. Subsequently it can contribute in the control programs for VL management and eradication.

## Supporting Information

S1 Fig Antibody capture ELISA with different dilutions of urine. Comparative optical density values of LAg-specific IgG antibodies of 5 VL patients, 2 healthy controls, 4 other diseases (2 malaria and 2 viral fevers) with different dilutions of urine reacted with 1.0 μg/well of LAg. Urine at 1:10 dilutions shows significant reactivity with VL while HC and OD samples have less reactivity.
(TIF)

S2 Fig Antibody-capture ELISA at different concentrations of LAg. Comparative optical density values of five VL patients (VL 1–5), two healthy controls (C1 and C2), two malaria (M1 and M2) and two viral fevers (V1 and V2) in 1:10 dilution of urine with different concentrations of LAg per well. LAg at 1.5μg/well shows clear difference in antibodies titre between VL with controls.
(TIF)

S3 Fig Dot blot assay at different concentrations of LAg. The sensitivity and specificity of LAg at different concentrations (2.0, 1.5, 1.0, and 0.5 μg/dot) tested by dot blot using urine samples. Strips 1 and 2 represent VL, strip 3 malaria, strip 4 viral fever, and strip 5 and 6 endemic and non-endemic healthy control urine samples, respectively. LAg at 1.5μg/well depicts visually clear difference between VL and controls.
(TIF)

S4 Fig Dot blot assay with different dilutions of urine. Results of dot blot with undiluted urine (1:0) and dilutions, 1:2, 1:5, 1:10, and 1:50. Strips 1 and 2 symbolize VL, strip 3 malaria, strip 4 viral fever, and strip 5 and 6 endemic and non-endemic healthy control urine samples, respectively. Urine at 1:5 dilutions best distinguishes VL from controls.
(TIF)

S5 Fig Dot blot assay with different blocking solutions. Dot blot reactivity when the membranes were blocked with 0.1% Tween-20, 1.5%, 3% and 5% BSA and 5% skimmed milk. In all the sets, strips 1 and 2 determine VL, strip 3 malaria, strip 4 viral fever, and strip 5 and 6 endemic and non-endemic healthy controls, respectively. Blocking with 5% BSA is optimal for clear reactivity of VL samples without any cross reactivity in controls.
(TIF)

S6 Fig Dot blot assay with anti-human IgG as control test. Results of dot blot coated with different dilutions of rabbit anti-human IgG antibody. In all the sets, strip 1 and 2 determines VL urine, strip 3 malaria urine, strip 4 viral fever urine, and strip 5 and 6 are endemic and non-endemic healthy controls urine, respectively. Antibodies at 1:20 dilutions show equal reactivity with all urine samples.
(TIF)

S7 Fig Prototype of a dipstick model. In a typical dipstick model; 1, support zone; 2, sample contact zone; 3, LAg; 4, test line; 5, anti-human IgG and 6, control line.
(TIF)

S8 Fig Immunological reactions for positive and negative urine sample in dipsticks. (TIF)

S1 Table Reactivity of pre and post treatment VL urine samples in urine based ELISA and dipstick assay. (DOCX)

S1 File Flow diagram of study subjects. (DOCX)

S2 File Clinical characteristics of participants. (DOCX)

S3 File STARD Checklist. (DOCX)

## References

[1] Alvar J, Velez ID, Bern C, Herrero M, Desjeux P, et al (2012) Leishmaniasis worldwide and global estimates of its incidence. PLoS One 7: e35671 10.1371/journal.pone.0035671 22693548 PMC3365071

[2] Gurunath U, Joshi R, Agrawal A, Shah V (2014) An overview of visceral leishmaniasis elimination program in India: a picture imperfect. Expert Rev Anti Infect Ther 12: 929–935. 10.1586/14787210.2014.928590 24930676

[3] Mondal S, Bhattacharya P, Ali N (2010) Current diagnosis and treatment of visceral leishmaniasis. Expert Rev Anti Infect Ther 8: 919–944. 10.1586/eri.10.78 20695748

[4] Pace D (2014) Leishmaniasis. J Infect 69 Suppl 1: S10–18. 10.1016/j.jinf.2014.07.016 25238669

[5] Sundar S, Chakravarty J (2012) Recent advances in the diagnosis and treatment of kala-azar. Natl Med J India 25: 85–89. 22686715

[6] Ejazi SA, Ali N (2013) Developments in diagnosis and treatment of visceral leishmaniasis during the last decade and future prospects. Expert Rev Anti Infect Ther 11: 79–98. 10.1586/eri.12.148 23428104

[7] Medley GF, Hollingsworth TD, Olliaro PL, Adams ER (2015) Health-seeking behaviour, diagnostics and transmission dynamics in the control of visceral leishmaniasis in the Indian subcontinent. Nature 528: S102–108. 10.1038/nature16042 26633763

[8] Boelaert M, Verdonck K, Menten J, Sunyoto T, van Griensven J, et al (2014) Rapid tests for the diagnosis of visceral leishmaniasis in patients with suspected disease. Cochrane Database Syst Rev: CD009135 10.1002/14651858.CD009135.pub2 24947503 PMC4468926

[9] Sivakumar R, Dey A, Sharma P, Singh S (2008) Expression and characterization of a recombinant kinesin antigen from an old Indian strain (DD8) of Leishmania donovani and comparing it with a commercially available antigen from a newly isolated (KE16) strain of L. donovani. Infect Genet Evol 8: 313–322. 10.1016/j.meegid.2008.02.004 18374635

[10] Pattabhi S, Whittle J, Mohamath R, El-Safi S, Moulton GG, et al (2010) Design, development and evaluation of rK28-based point-of-care tests for improving rapid diagnosis of visceral leishmaniasis. PLoS Negl Trop Dis 4 10.1371/journal.pntd.0000822 20856856 PMC2939046

[11] Abass E, Bollig N, Reinhard K, Camara B, Mansour D, et al (2013) rKLO8, a novel Leishmania donovani—derived recombinant immunodominant protein for sensitive detection of visceral leishmaniasis in Sudan. PLoS Negl Trop Dis 7: e2322 10.1371/journal.pntd.0002322 23875052 PMC3715527

[12] Bhargava P, Singh R (2012) Developments in diagnosis and antileishmanial drugs. Interdiscip Perspect Infect Dis 2012: 626838 10.1155/2012/626838 23118748 PMC3483814

[13] Mohapatra S, Samantaray JC, Ghosh A (2016) A Comparative Study of Serum, Urine and Saliva Using rk39 Strip for the Diagnosis of Visceral Leishmaniasis. J Arthropod Borne Dis 10: 87–91. 27047975 PMC4813396

[14] Motazedian M, Fakhar M, Motazedian MH, Hatam G, Mikaeili F (2008) A urine-based polymerase chain reaction method for the diagnosis of visceral leishmaniasis in immunocompetent patients. Diagn Microbiol Infect Dis 60: 151–154. 10.1016/j.diagmicrobio.2007.09.001 17931819

[15] Fisa R, Riera C, Lopez-Chejade P, Molina I, Gallego M, et al (2008) Leishmania infantum DNA detection in urine from patients with visceral leishmaniasis and after treatment control. Am J Trop Med Hyg 78: 741–744. 18458307

[16] Silva MA, Medeiros Z, Soares CR, Silva ED, Miranda-Filho DB, et al (2014) A comparison of four DNA extraction protocols for the analysis of urine from patients with visceral leishmaniasis. Rev Soc Bras Med Trop 47: 193–197. 10.1590/0037-8682-0233-2013 24861293

[17] da Silva MR, Brandao NA, Dorta ML, Fatima RD, Costa DL, et al (2015) Evaluation of an rK39-based immunochromatographic test for the diagnosis of visceral leishmaniasis in human saliva. Trop Biomed 32: 247–256. 26691253

[18] Vaish M, Singh OP, Chakravarty J, Sundar S (2012) rK39 antigen for the diagnosis of visceral leishmaniasis by using human saliva. Am J Trop Med Hyg 86: 598–600. 10.4269/ajtmh.2012.11-0127 22492142 PMC3403768

[19] Singh OP, Sundar S (2015) Developments in Diagnosis of Visceral Leishmaniasis in the Elimination Era. J Parasitol Res 2015: 239469 10.1155/2015/239469 26843964 PMC4710934

[20] Elmahallawy EK, Sampedro Martinez A, Rodriguez-Granger J, Hoyos-Mallecot Y, Agil A, et al (2014) Diagnosis of leishmaniasis. J Infect Dev Ctries 8: 961–972. 10.3855/jidc.4310 25116660

[21] Vallur AC, Tutterrow YL, Mohamath R, Pattabhi S, Hailu A, et al (2015) Development and comparative evaluation of two antigen detection tests for Visceral Leishmaniasis. BMC Infect Dis 15: 384 10.1186/s12879-015-1125-3 26395447 PMC4580298

[22] Abeijon C, Campos-Neto A (2013) Potential non-invasive urine-based antigen (protein) detection assay to diagnose active visceral leishmaniasis. PLoS Negl Trop Dis 7: e2161 10.1371/journal.pntd.0002161 23738023 PMC3667753

[23] Islam MZ, Itoh M, Shamsuzzaman SM, Mirza R, Matin F, et al (2002) Diagnosis of visceral leishmaniasis by enzyme-linked immunosorbent assay using urine samples. Clin Diagn Lab Immunol 9: 789–794. 10.1128/cdli.9.4.789-794.2002 12093674 PMC120024

[24] Islam MZ, Itoh M, Takagi H, Islam AU, Ekram AR, et al (2008) Enzyme-linked immunosorbent assay to detect urinary antibody against recombinant rKRP42 antigen made from Leishmania donovani for the diagnosis of visceral leishmaniasis. Am J Trop Med Hyg 79: 599–604. 18840751

[25] Mukhopadhyay D, Dalton JE, Kaye PM, Chatterjee M (2014) Post kala-azar dermal leishmaniasis: an unresolved mystery. Trends Parasitol 30: 65–74. 10.1016/j.pt.2013.12.004 24388776 PMC3919212

[26] Zijlstra EE (2016) Visceral leishmaniasis: a forgotten epidemic. Arch Dis Child 101: 561–567. 10.1136/archdischild-2015-309302 26895806

[27] Roychoudhury J, Sinha R, Ali N (2011) Therapy with sodium stibogluconate in stearylamine-bearing liposomes confers cure against SSG-resistant Leishmania donovani in BALB/c mice. PLoS One 6: e17376 10.1371/journal.pone.0017376 21423750 PMC3053369

[28] Mazumder S, Maji M, Das A, Ali N (2011) Potency, efficacy and durability of DNA/DNA, DNA/protein and protein/protein based vaccination using gp63 against Leishmania donovani in BALB/c mice. PLoS One 6: e14644 10.1371/journal.pone.0014644 21311597 PMC3032732

[29] Das A, Ali N (2014) Combining cationic liposomal delivery with MPL-TDM for cysteine protease cocktail vaccination against Leishmania donovani: evidence for antigen synergy and protection. PLoS Negl Trop Dis 8: e3091 10.1371/journal.pntd.0003091 25144181 PMC4140747

[30] Bhowmick S, Ravindran R, Ali N (2008) gp63 in stable cationic liposomes confers sustained vaccine immunity to susceptible BALB/c mice infected with Leishmania donovani. Infect Immun 76: 1003–1015. 10.1128/IAI.00611-07 18195029 PMC2258822

[31] Asad M, Bhattacharya P, Banerjee A, Ali N (2015) Therapeutic and immunomodulatory activities of short-course treatment of murine visceral leishmaniasis with KALSOME10, a new liposomal amphotericin B. BMC Infect Dis 15: 188 10.1186/s12879-015-0928-6 25884796 PMC4411769

[32] Saha S, Goswami R, Pramanik N, Guha SK, Saha B, et al (2011) Easy test for visceral Leishmaniasis and post-Kala-azar Dermal Leishmaniasis. Emerg Infect Dis 17: 1304–1306. 10.3201/eid1707.100801 21762596 PMC3381407

[33] Islam MZ, Itoh M, Mirza R, Ahmed I, Ekram AR, et al (2004) Direct agglutination test with urine samples for the diagnosis of visceral leishmaniasis. Am J Trop Med Hyg 70: 78–82. 14971702

[34] Singh OP, Sundar S (2012) Analysis of Total Urine Proteins: Towards A Non-Invasive Approach for Diagnosis of Visceral Leishmaniasis. J Mol Biomark Diagn 3 10.4172/2155-9929.1000131 23503981 PMC3595606

[35] Kumar V, Mishra M, Rajput SK, Bajpai S, Singh RK (2012) Detection and diagnostic applicability of human urinary kininogen in kala-azar patients. Parasitol Res 111: 1851–1855. 10.1007/s00436-012-2931-9 22562212

[36] Abeijon C, Kashino SS, Silva FO, Costa DL, Fujiwara RT, et al (2012) Identification and diagnostic utility of Leishmania infantum proteins found in urine samples from patients with visceral leishmaniasis. Clin Vaccine Immunol 19: 935–943. 10.1128/CVI.00125-12 22518013 PMC3370439

[37] Ghosh P, Bhaskar KR, Hossain F, Khan MA, Vallur AC, et al (2016) Evaluation of diagnostic performance of rK28 ELISA using urine for diagnosis of visceral leishmaniasis. Parasit Vectors 9: 383 10.1186/s13071-016-1667-2 27377266 PMC4932727

[38] Chakravarty J, Kumar S, Kumar R, Gautam S, Rai M, et al (2011) Evaluation of rk39 immunochromatographic test with urine for diagnosis of visceral leishmaniasis. Trans R Soc Trop Med Hyg 105: 537–539. 10.1016/j.trstmh.2011.05.008 21708392 PMC3163715

[39] Khan MG, Alam MS, Podder MP, Itoh M, Jamil KM, et al (2010) Evaluation of rK-39 strip test using urine for diagnosis of visceral leishmaniasis in an endemic area in Bangladesh. Parasit Vectors 3: 114 10.1186/1756-3305-3-114 21110875 PMC3003646

[40] Goswami RP, Das S, Ray Y, Rahman M (2012) Testing urine samples with rK39 strip as the simplest non-invasive field diagnosis for visceral leishmaniasis: an early report from eastern India. J Postgrad Med 58: 180–184. 10.4103/0022-3859.101378 23023349

[41] Singh D, Pandey K, Das VN, Das S, Verma N, et al (2013) Evaluation of rK-39 strip test using urine for diagnosis of visceral leishmaniasis in an endemic region of India. Am J Trop Med Hyg 88: 222–226. 10.4269/ajtmh.2012.12-0489 23149580 PMC3583308

[42] Chappuis F, Sundar S, Hailu A, Ghalib H, Rijal S, et al (2007) Visceral leishmaniasis: what are the needs for diagnosis, treatment and control? Nat Rev Microbiol 5: 873–882. 10.1038/nrmicro1748 17938629

[43] Reiter-Owona I, Rehkaemper-Schaefer C, Arriens S, Rosenstock P, Pfarr K, et al (2016) Specific K39 antibody response and its persistence after treatment in patients with imported leishmaniasis. Parasitol Res 115: 761–769. 10.1007/s00436-015-4801-8 26508007 PMC4722063

[44] Vallur AC, Hailu A, Mondal D, Reinhart C, Wondimu H, et al (2015) Specific antibody responses as indicators of treatment efficacy for visceral leishmaniasis. Eur J Clin Microbiol Infect Dis 34: 679–686. 10.1007/s10096-014-2282-9 25407374

[45] Hasnain MG, Ghosh P, Baker J, Mondal D (2014) An evaluation of the performance of direct agglutination test on filter paper blood sample for the diagnosis of visceral leishmaniasis. Am J Trop Med Hyg 91: 342–344. 10.4269/ajtmh.13-0530 24957542 PMC4125259

