# Supplementary figures and images for: Noninvasive Diagnosis of Visceral Leishmaniasis: Development and Evaluation of Two Urine-Based Immunoassays for Detection of Leishmania donovani Infection in India
Source: PLoS Negl Trop Dis. 2016 Oct 14;10(10):e0005035. doi: 10.1371/journal.pntd.0005035 (PMC5065134; doi:10.1371/journal.pntd.0005035)

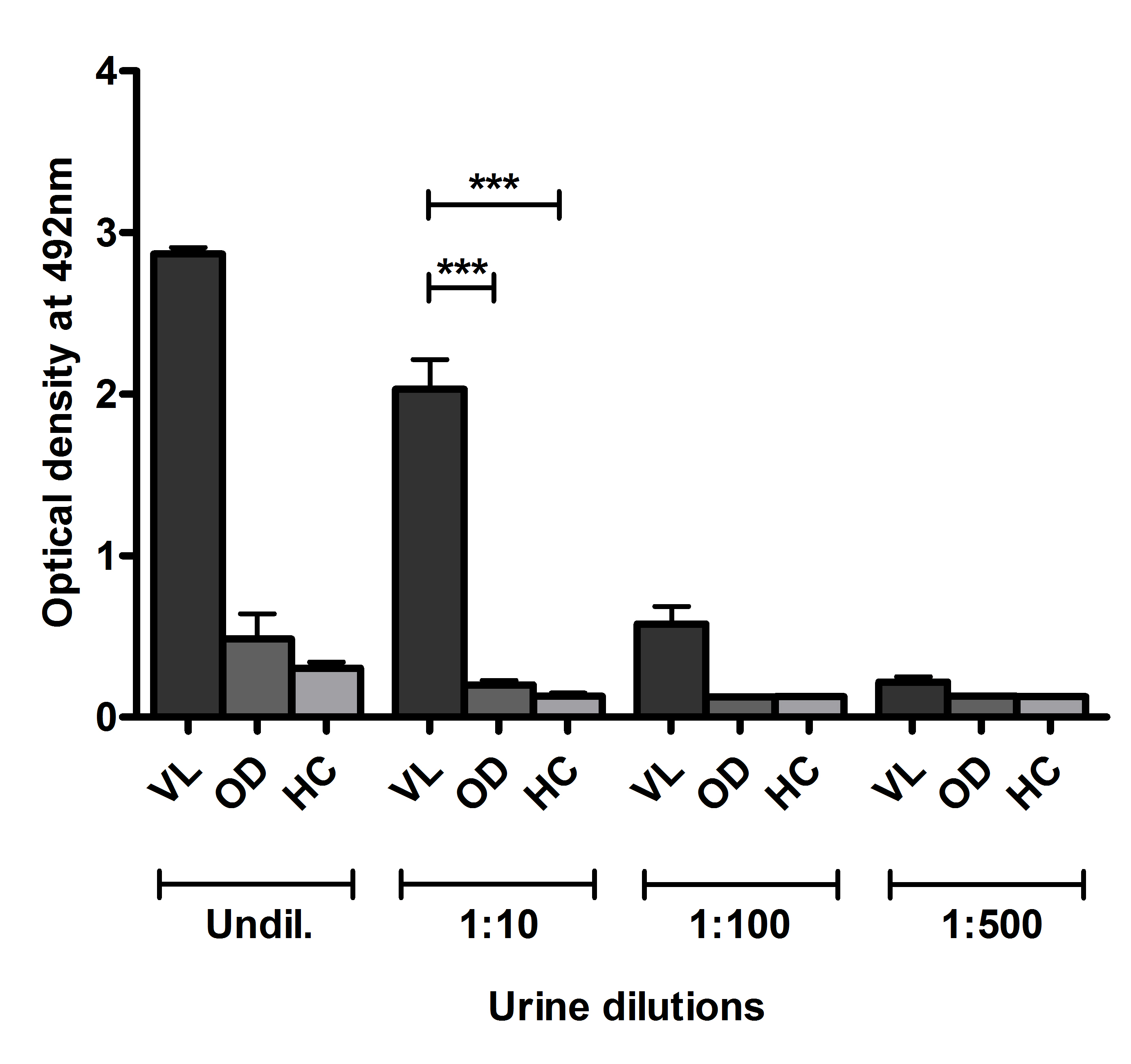

Supplement: S1 Fig — Comparative optical density values of LAg-specific IgG antibodies of 5 VL patients, 2 healthy controls, 4 other diseases (2 malaria and 2 viral fevers) with different dilutions of urine reacted with 1.0 μg/well of LAg. Urine at 1:10 dilutions shows significant reactivity with VL while HC and OD samples have less reactivity. (TIF) [file pntd.0005035.s001.tif]

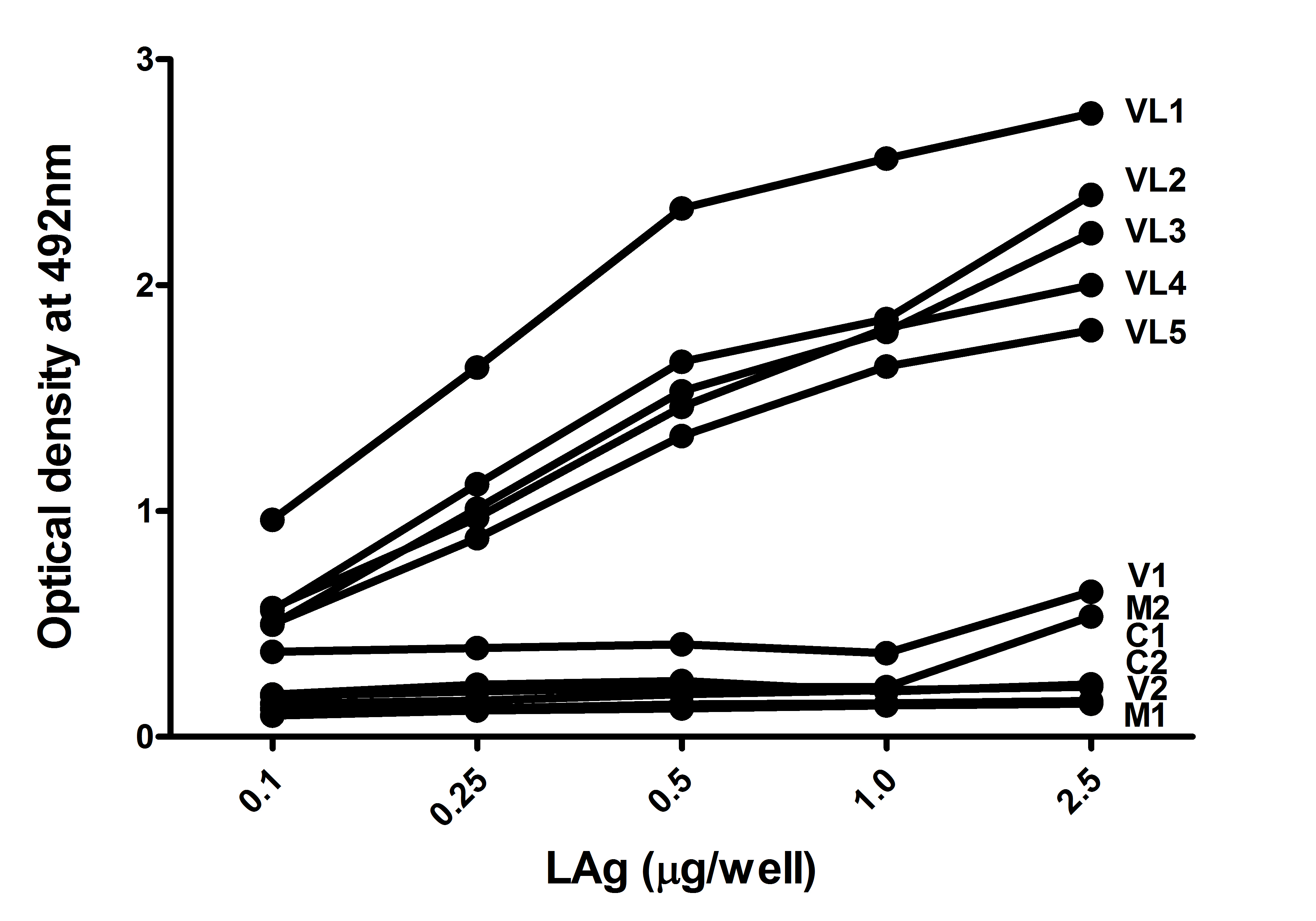

Supplement: S2 Fig — Comparative optical density values of five VL patients (VL 1–5), two healthy controls (C1 and C2), two malaria (M1 and M2) and two viral fevers (V1 and V2) in 1:10 dilution of urine with different concentrations of LAg per well. LAg at 1.5μg/well shows clear difference in antibodies titre between VL with controls. (TIF) [file pntd.0005035.s002.tif]

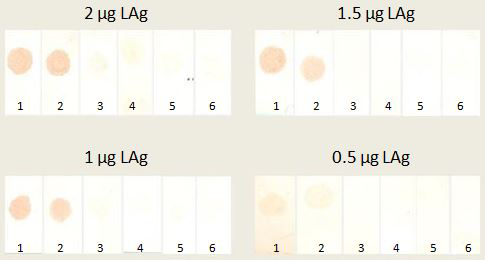

Supplement: S3 Fig — The sensitivity and specificity of LAg at different concentrations (2.0, 1.5, 1.0, and 0.5 μg/dot) tested by dot blot using urine samples. Strips 1 and 2 represent VL, strip 3 malaria, strip 4 viral fever, and strip 5 and 6 endemic and non-endemic healthy control urine samples, respectively. LAg at 1.5μg/well depicts visually clear difference between VL and controls. (TIF) [file pntd.0005035.s003.tif]

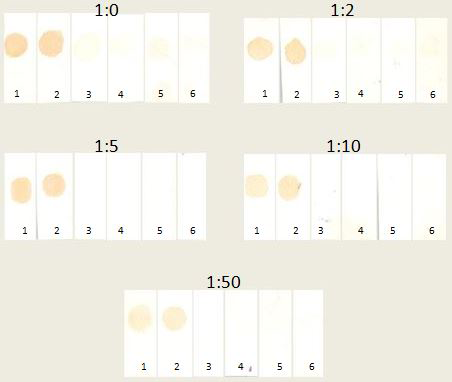

Supplement: S4 Fig — Results of dot blot with undiluted urine (1:0) and dilutions, 1:2, 1:5, 1:10, and 1:50. Strips 1 and 2 symbolize VL, strip 3 malaria, strip 4 viral fever, and strip 5 and 6 endemic and non-endemic healthy control urine samples, respectively. Urine at 1:5 dilutions best distinguishes VL from controls. (TIF) [file pntd.0005035.s004.tif]

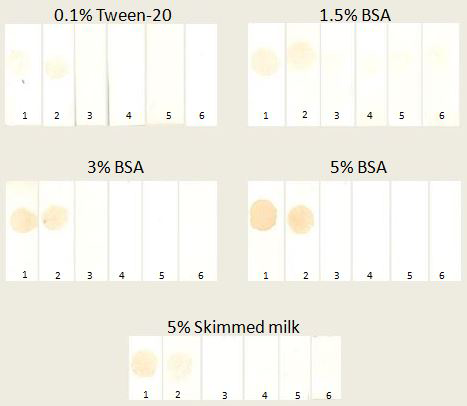

Supplement: S5 Fig — Dot blot reactivity when the membranes were blocked with 0.1% Tween-20, 1.5%, 3% and 5% BSA and 5% skimmed milk. In all the sets, strips 1 and 2 determine VL, strip 3 malaria, strip 4 viral fever, and strip 5 and 6 endemic and non-endemic healthy controls, respectively. Blocking with 5% BSA is optimal for clear reactivity of VL samples without any cross reactivity in controls. (TIF) [file pntd.0005035.s005.tif]

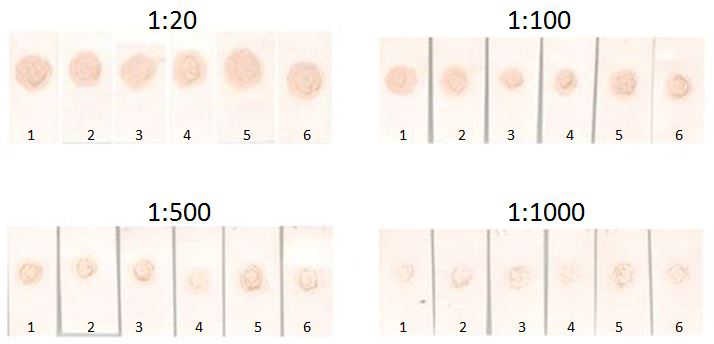

Supplement: S6 Fig — Results of dot blot coated with different dilutions of rabbit anti-human IgG antibody. In all the sets, strip 1 and 2 determines VL urine, strip 3 malaria urine, strip 4 viral fever urine, and strip 5 and 6 are endemic and non-endemic healthy controls urine, respectively. Antibodies at 1:20 dilutions show equal reactivity with all urine samples. (TIF) [file pntd.0005035.s006.tif]

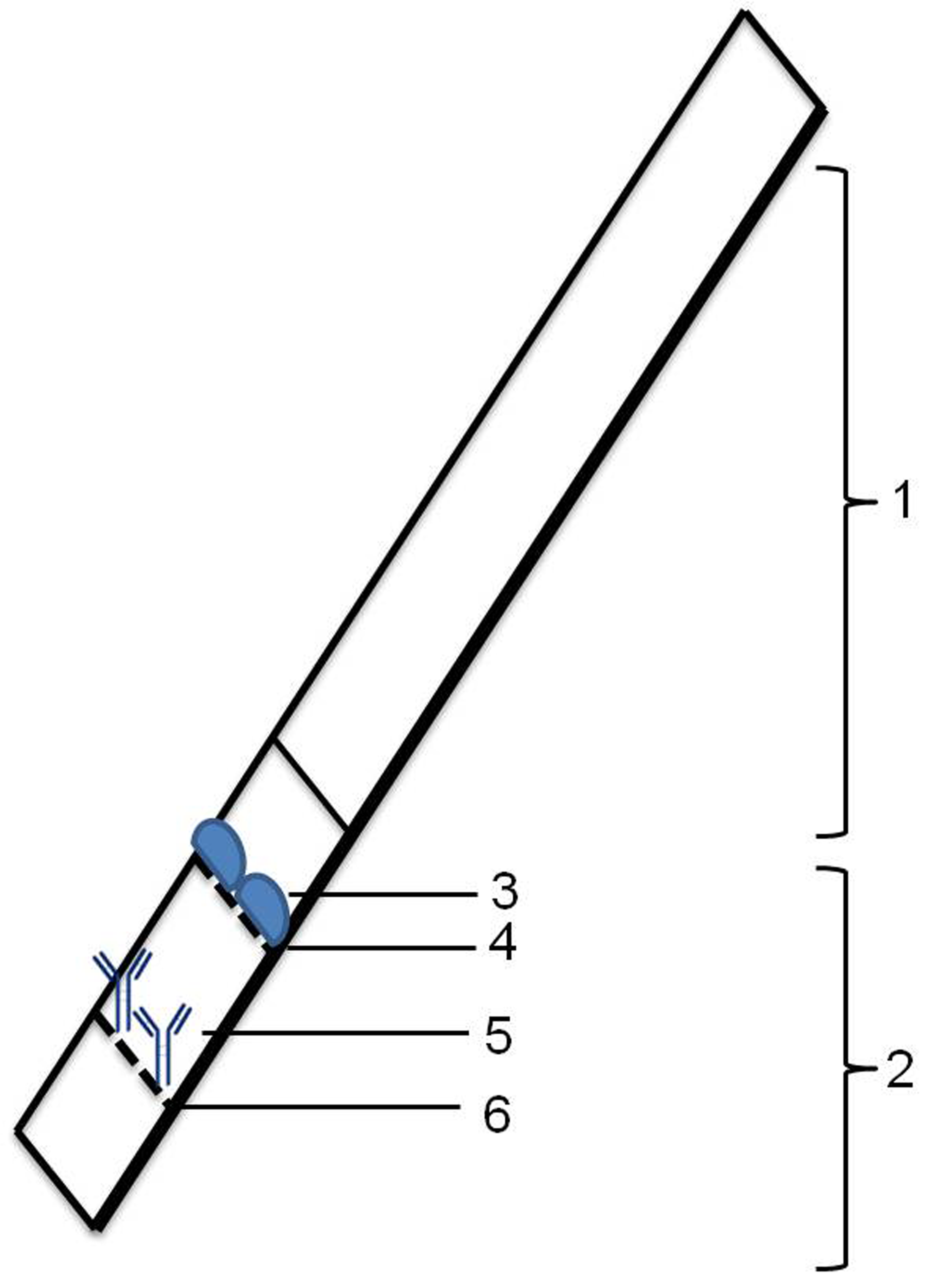

Supplement: S7 Fig — In a typical dipstick model; 1, support zone; 2, sample contact zone; 3, LAg; 4, test line; 5, anti-human IgG and 6, control line. (TIF) [file pntd.0005035.s007.tif]

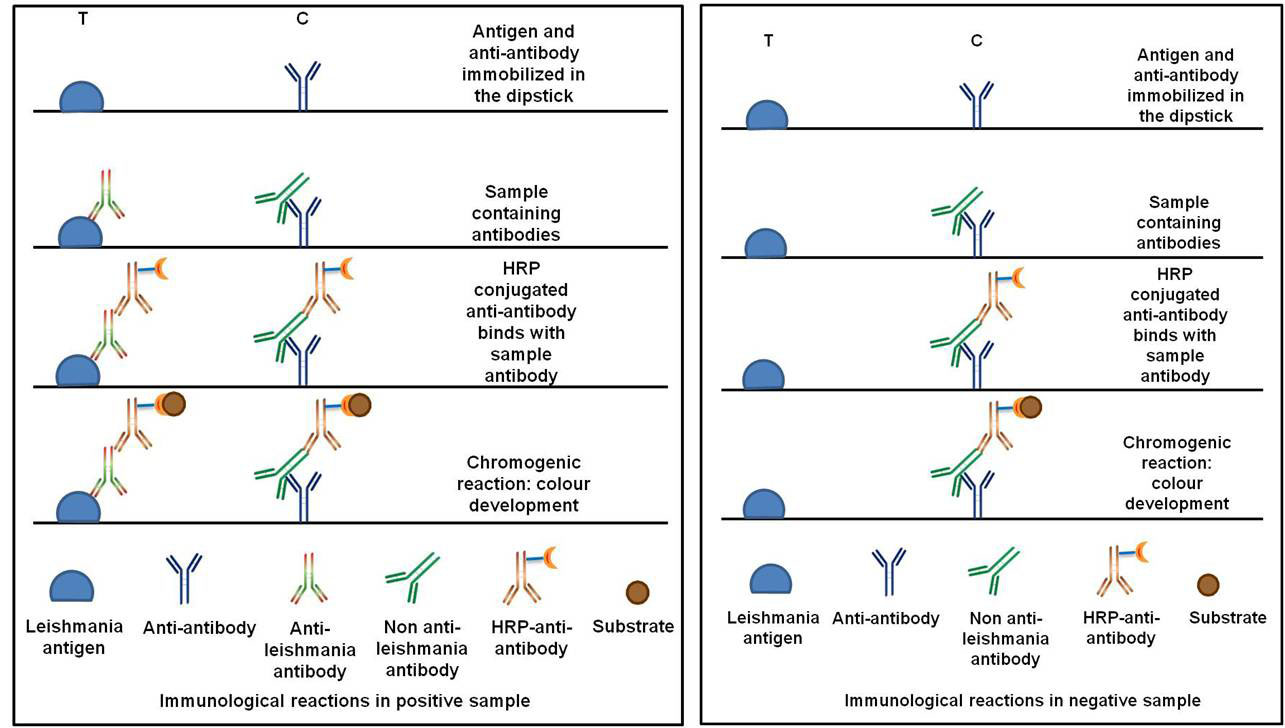

Supplement: S8 Fig — (TIF) [file pntd.0005035.s008.tif]
