# Supplementary material for: Noninvasive Diagnosis of Visceral Leishmaniasis: Development and Evaluation of Two Urine-Based Immunoassays for Detection of Leishmania donovani Infection in India
Source: PLoS Negl Trop Dis. 2016 Oct 14;10(10):e0005035. doi: 10.1371/journal.pntd.0005035 (PMC5065134; doi:10.1371/journal.pntd.0005035)
Supplement: S1 Table — (DOCX) [file pntd.0005035.s009.docx]

|  | **Urine-ELISA** | | **Urine-dipstick** | |
| --- | --- | --- | --- | --- |
| **VL (n=18)** | **Pre treatment**  **(day 0)** | **Post treatment (day >180)** | **Pre treatment**  **(day 0)** | **Post treatment (day >180)** |
| **Positives** | 18 | 2 | 18 | 2 |
| **Negatives** | 0 | 16 | 2 | 16 |

**Reactivity of pre and post treatment VL urine samples in urine-based ELISA and dipstick assay.**
