# Supplementary material for: Noninvasive Diagnosis of Visceral Leishmaniasis: Development and Evaluation of Two Urine-Based Immunoassays for Detection of Leishmania donovani Infection in India
Source: PLoS Negl Trop Dis. 2016 Oct 14;10(10):e0005035. doi: 10.1371/journal.pntd.0005035 (PMC5065134; doi:10.1371/journal.pntd.0005035)
Supplement: S1 File — (DOCX) [file pntd.0005035.s010.docx]

**Annexure-1**

**Flow diagram of participants in the study**
