# Supplementary material for: Noninvasive Diagnosis of Visceral Leishmaniasis: Development and Evaluation of Two Urine-Based Immunoassays for Detection of Leishmania donovani Infection in India
Source: PLoS Negl Trop Dis. 2016 Oct 14;10(10):e0005035. doi: 10.1371/journal.pntd.0005035 (PMC5065134; doi:10.1371/journal.pntd.0005035)
Supplement: S2 File — (DOCX) [file pntd.0005035.s011.docx]

**Annexure-2**

**Clinical characteristics of participants (Mean±SD)**

| Characteristics | VL (n=97) | >180 days VL (n=18) | PKDL (n=14) | Controls (n=75) |
| --- | --- | --- | --- | --- |
| Age (yrs) | 25.9± 1.66 | 31.2±3.54 | 22.3±4.49 | 26.1±1.90 |
| Male/female | 64/33 | 14/4 | 9/5 | 47/28 |
| Body weight (Kg) | 39.1±1.56 | 40.7±3.43 | 33.0±3.75 | 38.9±1.83 |
| Spleen size (cm) | 8.47±0.31 | - | - | - |
| Fever duration (months) | 4.32±0.39 | - | - | - |
| Haemoglobin (g/dl) | 7.9±0.16 | - | - | - |
| White blood cells count (cells/µl) | 2930±840 | - | - | - |
| Platelet count (1X 10^3^/ml) | 143±7.5 | - | - | - |
