# Supplementary material for: Noninvasive Diagnosis of Visceral Leishmaniasis: Development and Evaluation of Two Urine-Based Immunoassays for Detection of Leishmania donovani Infection in India
Source: PLoS Negl Trop Dis. 2016 Oct 14;10(10):e0005035. doi: 10.1371/journal.pntd.0005035 (PMC5065134; doi:10.1371/journal.pntd.0005035)
Supplement: S3 File — (DOCX) [file pntd.0005035.s012.docx]

STARD CHECKLIST

*(version 2015)*

| **Section and Topic** | **No.** |  |
| --- | --- | --- |
| TITLE/ABSTRACT/  KEYWORDS |  |  |
|  | 1 | *Identification as a study of diagnostic accuracy using at least one measure of accuracy (such as sensitivity, specificity, predictive values, or AUC):*  Study state development and evaluation of urine-based ELISA and dipstick test as noninvasive diagnostics for VL and PKDL. |
| ABSTRACT |  |  |
|  | 2 | *Structured summary of study design, methods, results, and conclusions (for specific guidance, see STARD for Abstracts):*  Identify the article as a study of diagnostic accuracy using measures of sensitivity, specificity and AUC.  Provides summary of study design, results and conclusion |
| INTRODUCTION |  |  |
|  | 3 | *Scientific and clinical background, including the intended use and clinical role of the index test:*  State the background of the work such as routine diagnostics and other similar test available |
|  | 4 | *Study objectives and hypotheses:*  Study objectives such as development of noninvasive test and its comparison with reference test of tissue aspiration and with rK39 strip test. |
| METHODS |  |  |
| *Study design* | 5 | *Whether data collection was planned before the index test and reference standard were performed (prospective study) or after (retrospective study):*  This study is a prospective study which were planned before the reference and index tests were performed |
| *Participants* | 6 | *Eligibility criteria:*  Confirmed VL patients of both sexes living in and around the endemic area and ready to give consent. |
|  | 7 | *On what basis potentially eligible participants were identified (such as symptoms, results from previous tests, inclusion in registry):*  Participants were recruited based on clinical symptoms, other test results and tissue aspiration with rK39 rapid tests. Urine samples were collected after disease confirmation. |
|  | 8 | *Where and when potentially eligible participants were identified (setting, location and dates):*  Urine samples of confirmed cases and other diseases were provided by three centers, Patna, Muzaffarpur and Kolkata where reference test were done. Urine samples of healthy participants were collected in the institute, IICB where index test were conducted. |
|  | 9 | *Whether participants formed a consecutive, random or convenience series Test methods:*  Reference test was not performed separately for the study. Tissue aspiration and rK39 strip tests were routinely performed for the diagnosis of VL. |
| *Test methods* | 10a | *Index test, in sufficient detail to allow replication:*  Urine-based ELISA and dipstick test were developed and evaluated for diagnosis of VL and PKDL. Details available in the manuscript. |
|  | 10b | *Reference standard, in sufficient detail to allow replication:*  Reference standard is splenic or bone marrow aspirations for detection of *L. donovani* parasite in the tissues. |
|  | 11 | *Rationale for choosing the reference standard (if alternatives exist):*  Gold standard for the diagnosis of VL is tissue aspiration. This is the first line of diagnostics routinely used by the practitioners. |
|  | 12a | *Definition of and rationale for test positivity cut-offs or result categories of the index test, distinguishing pre-specified from exploratory:*  Diagnostic accuracy of the index test was calculated using Graphpad prism software and it was not pre-specified as the index test is developed during the study. |
|  | 12b | *Definition of and rationale for test positivity cut-offs or result categories of the reference standard, distinguishing pre-specified from exploratory:*  Reference test were used with known cases thus sensitivities and specificities of the tests were pre-determined. |
|  | 13a | *Whether clinical information and reference standard results were available to the performers/readers of the index test*  Yes, as index tests were performed with samples which were performed with reference test. |
|  | 13b | *Whether clinical information and index test results were available to the assessors of the reference standard analysis:*  Yes |
|  | 14 | *Methods for estimating or comparing measures of diagnostic accuracy.*  ROC curve were plotted and sensitivity, specificity, cut-offs and AUC of the tests were calculated. |
|  | 15 | *How indeterminate index test or reference standard results were handled*  Indeterminate reference test were discarded from the study where as uncertain results of index tests were re-evaluated. |
|  | 16 | *How missing data on the index test and reference standard were handled*  NA |
|  | 17 | *Any analyses of variability in diagnostic accuracy, distinguishing pre-specified from exploratory:*  No |
|  | 18 | *Intended sample size and how it was determined:*  Anticipation of 95% sensitivity with 95% confidence interval a 0.8 power 114 samples were estimated, however, we successfully evaluated 97 samples. |
| RESULTS |  |  |
| *Participants* | 19 | *Flow of participants, using a diagram.*  Presented in Annexure-1 |
|  | 20 | *Baseline demographic and clinical characteristics of participants:*  Presented in Annexure -2 |
|  | 21a | *Distribution of severity of disease in those with the target condition*  Not determined |
|  | 21b | *Distribution of alternative diagnoses in those without the target condition*  Performed with malaria, viral fever and typhoid and tuberculosis. |
|  | 22 | *Time interval and any clinical interventions between index test and reference standard*  Both the tests were performed before the treatment started, reference test followed by index test with 48 hrs. |
| *Test results* | 23 | *Cross tabulation of the index test results (or their distribution) by the results of the reference standard*  Results of both the index test urine-based ELISA and dipstick test were tabulated in Table 1 with rK39 strip test result. Participants who were tested positive in the reference test were only included thus not compared with the index tests. |
|  | 24 | *Estimates of diagnostic accuracy and their precision (such as 95% confidence intervals)*  More than 95% sensitivity and specificity were estimated for the test with 95% confidence interval and 0.8 power of the study. |
|  | 25 | *Any adverse events from performing the index test or the reference standard*  No |
| DISCUSSION |  |  |
|  | 26 | *Study limitations, including sources of potential bias, statistical uncertainty, and generalisability:*  Study was conducted with confirmed cases in reference standard test. Therefore reference test was not blind. |
|  | 27 | *Implications for practice, including the intended use and clinical role of the index test;*  Discussed in discussion section. |
| OTHER INFORMATION |  |  |
|  | 28 | *Registration number and name of registry:*  NA |
|  | 29 | *Where the full study protocol can be accessed:*  YES |
|  | 30 | *Sources of funding and other support; role of funders*  This study was funded by Council of Scientific and Industrial Research, India. |
